# A Fall Worth It: Cutaneous Metastatic Deposit of a Distant Colorectal Cancer With Fistula-in-Ano

**DOI:** 10.7759/cureus.9979

**Published:** 2020-08-24

**Authors:** Sarit Badiani, Edward Cooper, Christophe R Berney

**Affiliations:** 1 Department of Surgery, Bankstown Hospital, Sydney, AUS

**Keywords:** metastasis, perianal fistula, cutaneous metastasis

## Abstract

A seeded fistula-in-ano from a synchronous colon cancer is rare. We report an unconventional case of a 70-year old male who presented with an incidental post-traumatic perianal cutaneous lump following a fall. Lesion biopsy confirmed the presence of a cutaneous malignant deposit. Further workup confirmed the diagnosis of upper rectal adenocarcinoma associated with a fistula-in-ano. The patient underwent long-course neoadjuvant chemoradiotherapy, followed by an “en bloc” laparoscopic abdominoperineal and extended fistula tract resection without complication. This case highlights a rare case of post-traumatic synchronous upper rectal cancer seeding into a low fistula-in-ano tract associated with a cutaneous perianal metastatic deposit.

## Introduction

A handful of studies have reported primary colon cancer arising from a chronic anal fistula, but distal implantation from a proximal adenocarcinoma into an anal fistula remains a rare condition [1]. Cancer implantation is usually suspected when carcinoma originating in an anal fistula has similarity of histological findings between the primary colorectal cancer and the metastatic lesion [2]. We present a very unusual case of post-traumatic synchronous upper rectal cancer, seeding into a low fistula-in-ano tract and associated with distant cutaneous perianal metastatic deposit.

## Case presentation

A 70-year-old male presented to his family physician with a tender subcutaneous mass in the left perianal region, following a fall onto his buttock. The mass was initially thought to be an infected hematoma and drainage was attempted, revealing a solid mass with a necrotic component. This was biopsied and unexpectedly confirmed the presence of a metastatic adenocarcinoma deposit.

The patient denied any recent change in bowel habit or mucous discharge but recalled occasional altered blood movements. He never had any previous colonoscopy. Clinical examination was normal. Locally, there was a firm and tender perianal scar situated at 5 o’clock in gynecological position approximately 2 cm from the anal verge. Rectal examination was normal. Blood tests showed a mild anemia (Hb 109 g/L) and carcinoembryonic antigen (CEA) level was raised at 33 mg/L. 

**Figure 1:**
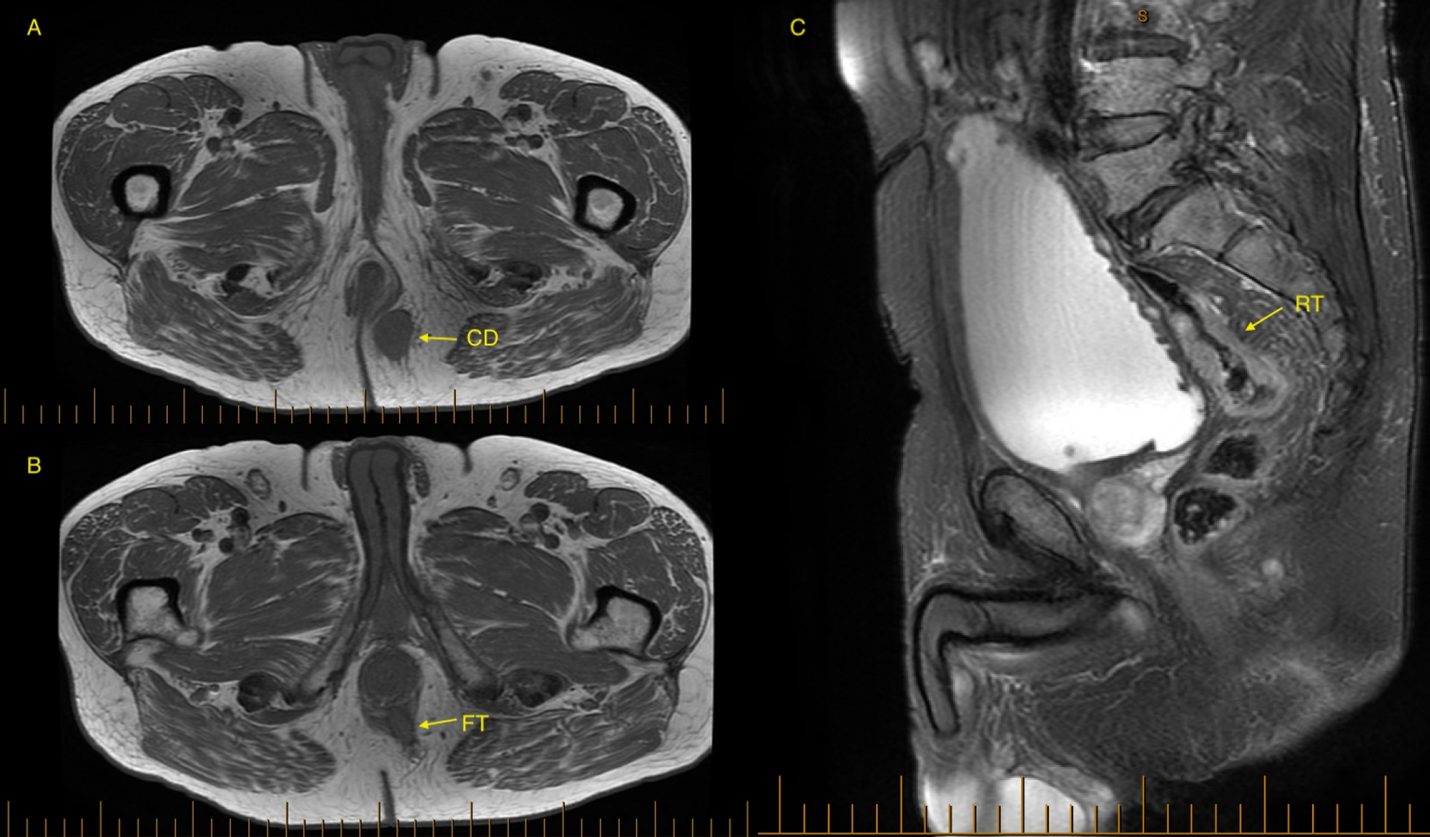
Axial and saggital views of MRI demonstrate cutaneous metastatic deposit (A), fistula-in-ano (B) and upper rectal tumor (C). CD, cutaneous deposit; FT, fistula tract; RT, rectal tumor

Colonoscopy identified a fungating non-obstructing mass in the proximal rectum, and biopsies confirmed the diagnosis of moderately differentiated adenocarcinoma. Staging CT did not show any evidence of distant metastases but the presence of a left-sided perianal soft tissue mass abutting the anal canal wall. This spiculated lesion measured 31 × 24 × 34 mm on complementary MRI and was incidentally associated with the presence of a fistulous anal tract extending superiorly to the external anal sphincter (Figure 1). An apple core lesion situated at the rectosigmoid junction was also noted and associated with prominent presacral lymph nodes (Figure 1).

**Figure 2:**
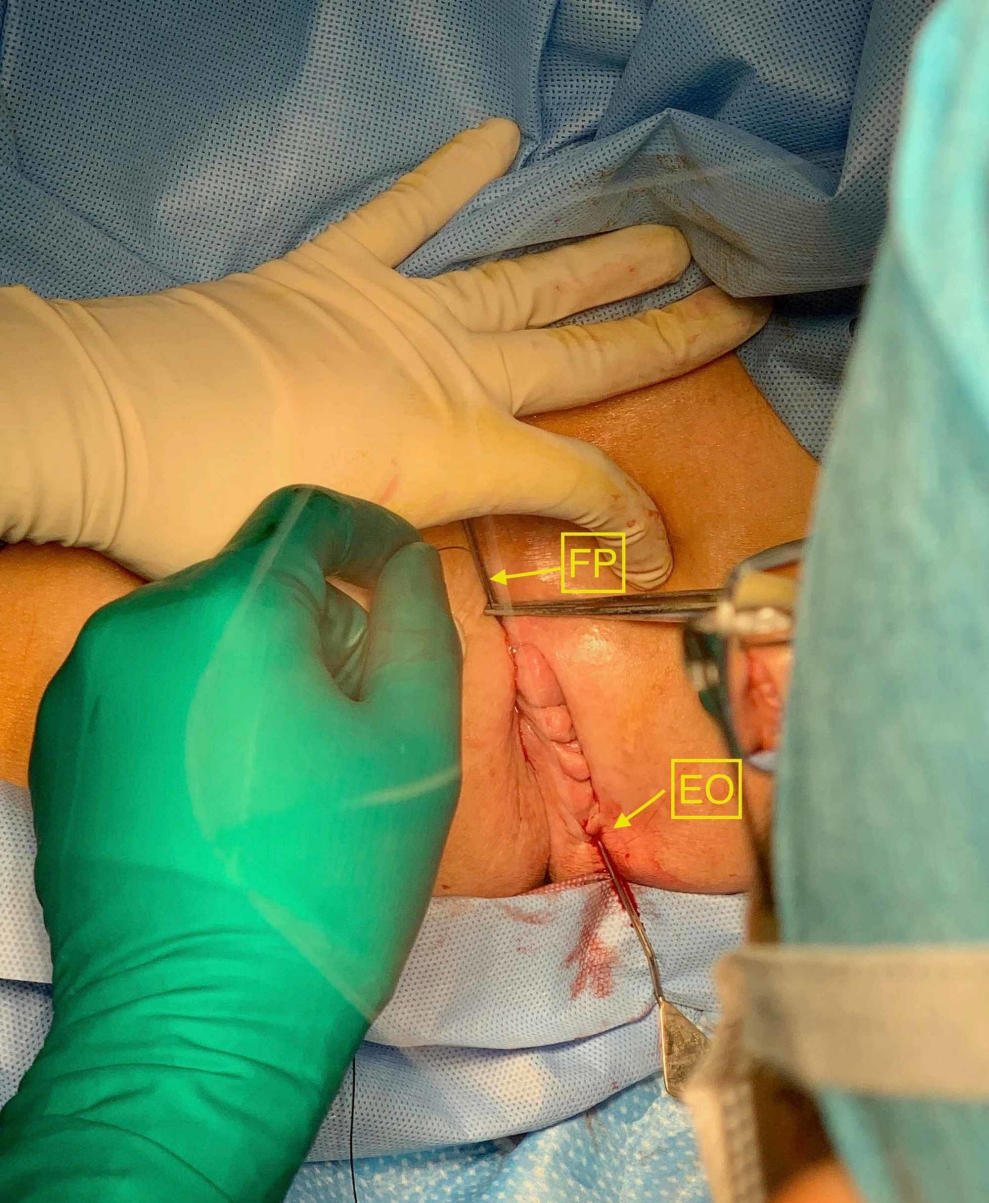
Intraoperative photograph demonstrating insertion of fistula probe and insertion of seton to guide en bloc resection. EO, external opening of fistula tract; FP, fistula probe

Following presentation at our local multidisciplinary oncology meeting, he was referred for neoadjuvant chemoradiotherapy (CRT) consisting of 50 Gy in 25 fractions with concurrent oral capecitabine over a five-week period. He tolerated this regimen well with minimal toxicity, and progress MRI confirmed a good radiological response (MRI tumor regression grade 3). The patient underwent elective "en bloc” laparoscopic abdominoperineal and extended fistula tract resection, 10-week after completion of his CRT. The initial perineal component of the operation consisted of probe insertion into the anal fistula tract and placement of a temporary seton to guide dissection and ensure adequate radial margins (Figure 2). The patient made an uneventful recovery and was discharged home four days postoperative.

**Figure 3:**
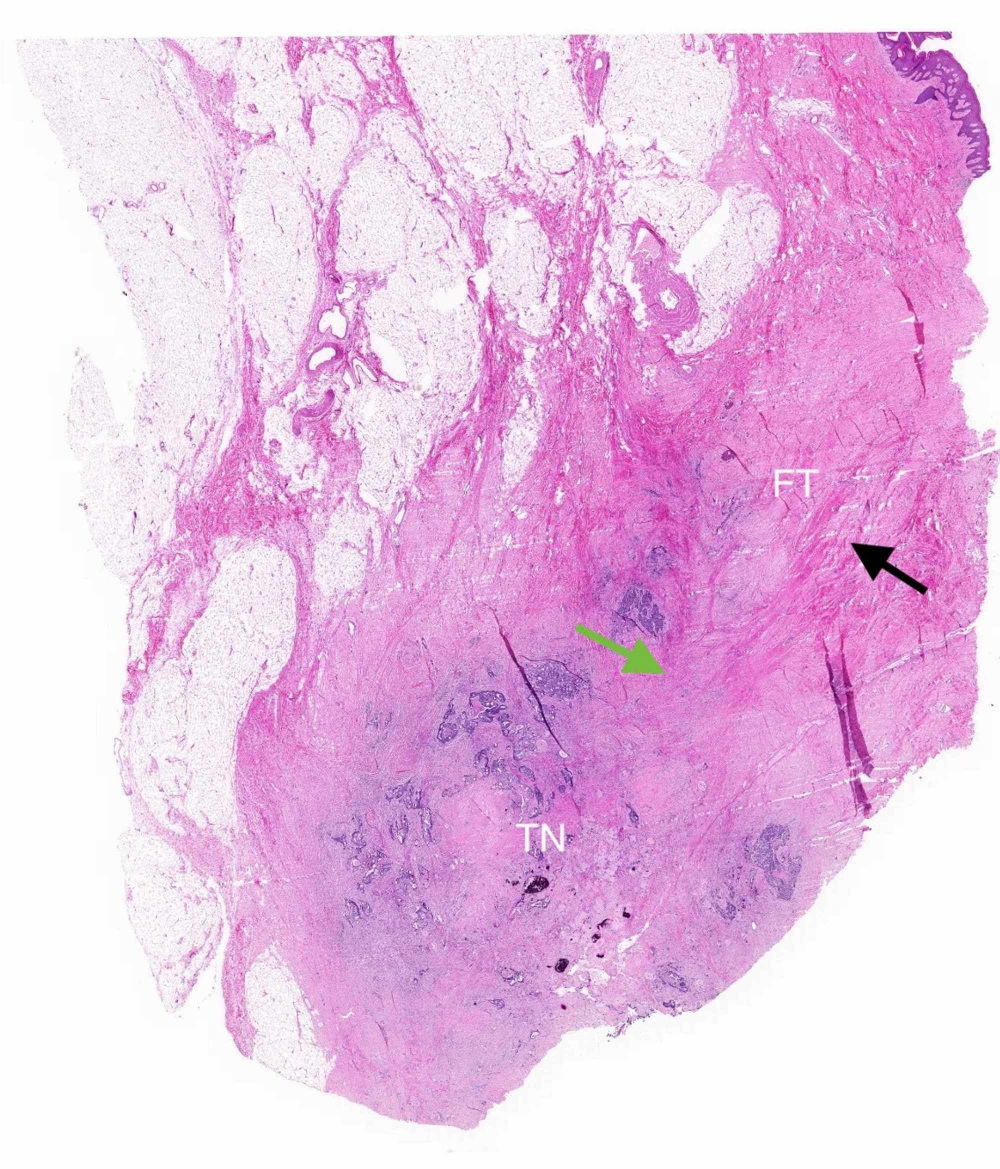
Histopathology confirming adenocarcinoma on tumor nodule and associated fistula tract. The black arrow indicates fistula tract. The green arrow showing radiation-induced fibrosis. FT, fistula tract; TN, tumor nodule

Final histopathology confirmed a moderately differentiated upper rectal cancer with a residual dimension of 11 mm x 10 mm, invading the muscularis propria and into the perirectal fat (ypT3). The fistula tract was associated with acute inflammation in close proximity to the 20 mm metastatic perianal connective tissue deposit, consisting of pleomorphic cells and luminal necrosis. The morphology was similar to the primary tumor (Figure 3). Excision was complete, and all lymph nodes were negative.

## Discussion

Cutaneous deposit from colorectal cancer typically presents as delayed post-surgical recurrence rather than a synchronous lesion at initial presentation. Most frequent locations include the abdomen, extremities and chest. Appearances range from single, subdermal flesh-colored to multiple, firm, violaceous nodules [3]. Mechanisms of cutaneous spread include lymphatic infiltration, intravascular dissemination and direct interstitial infiltration of the dermis [4]. Cutaneous perineal deposits from a rectal primary are exceedingly rare with 29 cases previously reported. In only eight of those, a cancer secondary deposit was the first sign of an underlying malignancy, such as in this case [5]. 

The first report of implantation metastasis from colorectal adenocarcinoma into a fistula-in-ano was reported by Guiss in 1954 [6]. Since then, only 30 similar cases have been reported to date [7,8]. The proposed mechanism of seeding is thought to be related to shedding of neoplastic cells into the intestinal lumen and subsequently implanting into injured mucosa [9]. This hypothesis has been supported with reports of exfoliated cancer cells implanting in distal locations such as the staple line and post-hemorrhoidectomy wounds resulting in tumor recurrence [10,11]. This is an important finding as preoperative identification may change the operative approach, resulting in a more radical abdominoperineal resection (APR) such as in our case. The literature appears divided between surgeons who prefer radical APR and others who opt for sphincter-sparing surgery. We did not biopsy the fistula tract prior to surgery as this would not have altered the planned definitive management. Final histopathology findings of the resected APR specimen demonstrated that the fistula-in-ano was intimately associated with the metastatic cutaneous deposit and characterized by significant radiation-induced fibrosis, but no cancer cells were identified in the fistula tract. The intimate association of the cutaneous lesion and the fistula tract is most likely from seeding through the tract to the perianal skin.

Due to the infrequency of this type of pathology, there is no consensus to the optimal management strategy. Treatment can consist of radiotherapy alone, neoadjuvant CRT prior to APR or anecdotally, local resection. It is important that each approach is tailored to the individual, taking into account patient comorbidities and global psychological preparedness, as well as tumor factors including location, size, dissemination and technical feasibility.

## Conclusions

This is a rare case of a perineal cutaneous colorectal metastatic deposit associated with a perianal fistula, which was incidentally diagnosed following a traumatic event. A fall worth it as the patient underwent a curative surgical treatment with significant chance of long-term cancer-free survival.
